# Management Options for Traumatic Posterior Sternoclavicular Joint Dislocation: A Narrative Review with a Single Institution’s Experience

**DOI:** 10.3390/jcm13185428

**Published:** 2024-09-13

**Authors:** Corrado Ciatti, Virginia Masoni, Pietro Maniscalco, Chiara Asti, Calogero Puma Pagliarello, Gianfilippo Caggiari, Marco Pes, Fabrizio Rivera, Fabrizio Quattrini

**Affiliations:** 1 Department of Orthopedics and Traumatology, Guglielmo da Saliceto Hospital, AUSL Piacenza, 29121 Piacenza, Italy; p.maniscalco@ausl.pc.it (P.M.); c.asti@studenti.uniss.it (C.A.); c.pumapagliarello@ausl.pc.it (C.P.P.); fabrizioquattrini@yahoo.it (F.Q.); 2 Department of Orthopedics and Traumatology, University of Parma, 43121 Parma, Italy; 3 Department of Orthopedics and Traumatology, University of Turin, Via Zuretti, 29, 10126 Turin, Italy; virginia.masoni@unito.it; 4 Department of Orthopedics and Traumatology, University of Sassari, 07100 Sassari, Italy; gianfilippocaggiari@gmail.com (G.C.); marco92pes@gmail.com (M.P.); 5 Department of Orthopedics and Traumatology, Ospedale SS Annunziata, ASL CN1, Via Ospedali, 9, 12038 Savigliano, Italy; rivgio@libero.it

**Keywords:** SCJ, posterior dislocation, biomechanics, figure-of-eight, semitendinosus

## Abstract

**Background:** Posterior sternoclavicular joint (SCJ) dislocations are rare events that can evolve into real emergencies due to the vital structures in the mediastinum. When closed reduction maneuvers fail, open SCJ reconstruction becomes mandatory, with literature proposing several stabilization techniques that either preserve or remove the SCJ’s mobility. This study is a narrative review of the most recent literature regarding posterior trauma to the SCJ along with a single institution’s experience of this pathology, managed either conservatively or surgically, with a figure-of-eight autologous semitendinosus graft in case of closed reduction failure. **Methods:** This article provides an overview of posterior traumatic SCJ dislocation, and it describes five cases of patients managed for traumatic posterior SCJ dislocation treated either conservatively or surgically with a figure-of-eight semitendinosus tendon autograft reinforced with high-strength suture tape. A comparison with the most recent literature is performed, focusing on biomechanics. **Results:** The demographics, the mechanism of injury, the management algorithm and the surgical strategy align with the most recent literature. Despite the final treatment, at one year of follow-up, the ROM was restored with full strength throughout the range of motion of the shoulder with no neurological deficits. The reduced joint successfully healed in imaging, and patients returned to their daily lives. The surgical site wounds and donor harvest sites were perfectly healed. **Conclusions:** Although recent recommendations for treating posterior traumatic SCJ dislocation have advanced, no universally accepted method of stabilization exists, and the surgical strategy is generally entrusted to the surgeon’s experience. The literature still increasingly supports figure-of-eight ligament reconstruction with a biological or synthetic graft. This work further implements the literature by reporting good outcomes at follow-up.

## 1. Introduction

Sternoclavicular joint (SCJ) injuries represent 3% of all shoulder-girdle lesions and less than 1% of all dislocations [1,2,3,4]. Traumatic dislocations are more likely to occur in young, active men after high-energy trauma, such as sports injuries, road traffic accidents and falls [1,2,3,5,6,7].

Dislocations most commonly occur anteriorly due to the SCJ’s anatomy, which includes a stout posterior capsular ligament and the costoclavicular ligament [1,8]. Nonetheless, posterior SCJ dislocation may lead to life-threatening consequences due to vital structures in the mediastinum [7,8,9].

For this reason, a high index of suspicion and immediate identification is necessary, with a missing rate of 25% at initial presentation [1,2,3,7,8,9,10,11,12,13]. The patient most often complains of neck and shoulder pain, which increases with movement of the ipsilateral girdle, with a possible palpable defect and deformity of the SCJ [1,3].

While radiography, in a serendipity view, is used, a CT scan is the preferred imaging modality to diagnose dislocation and assess the mediastinal structures [2,14].

Another classification is acute or chronic SCJ dislocation, with the chronic ones resulting from missed initial diagnosis, delayed clinical presentation and recurrent dislocation after closed reduction performed in the acute setting [14,15,16].

Despite the normal range of motion (ROM) of SCJ being narrow, this joint impacts the scapulothoracic, the glenohumeral rhythm, and the ROM of the shoulder girdle, being the only true connection between the axial skeleton and the upper limb [1,2,3].

The literature has developed several recommendations concerning the management of posterior SCJ dislocations [1,2,3]. A closed reduction is attempted in the first 48 h, followed by a subsequent imaging re-evaluation [1,2,3]. In case of failure or recurrent chronic instability, an open reduction is necessary, and, in this case, SCJ reconstruction is suggested [1,17]. However, no universal guidelines exist concerning the surgical strategy that should be adopted to stabilize this joint [3,18]. The review by Kendal et al. [2] and the most recent literature [1,3], based on the biomechanical study by Spencer and Kuhn in 2004 [19], support reconstruction with tendon grafts, especially with a figure-of-eight configuration [1,2,3]. Pins and Kirschner wires should be avoided due to possible migration in the vital structures of the mediastinum, as well as ORIF with plates and screws due to the necessity of their removal to restore motion [1,2,3].

This study provides a comprehensive overview on posterior traumatic SCJ dislocation. Management algorithms and outcomes are described. Moreover, it reports the experience from a single institution by describing five cases of this rare pathology managed either conservatively or surgically. Specifically, it focuses on the adopted surgical strategy, which consists of the reconstruction with a figure-of-eight autologous semitendinosus graft. Preoperative and intraoperative details are reported. A comparison with the most recent literature concerning the management methods is performed, with a complete explanation of the importance of the anatomy and biomechanics as the leading factors guiding authors to adopt this surgical technique.

## 2. Methods, Study Design and Surgical Technique

A comprehensive narrative review of the current literature on posterior traumatic SCJ dislocations was conducted taking into account the epidemiology, the management and the adopted surgical strategy for this pathology.

A retrospective analysis of all orthopedic consults occurring at the Emergency Department (ED) of the Guglielmo da Saliceto Hospital in Piacenza in Italy was performed to identify episodes of posterior sternoclavicular joint dislocation. Patient data were extracted from the database of the ED. The analyzed time frame was from January 2020 to December 2023.

**Scheme 1:**
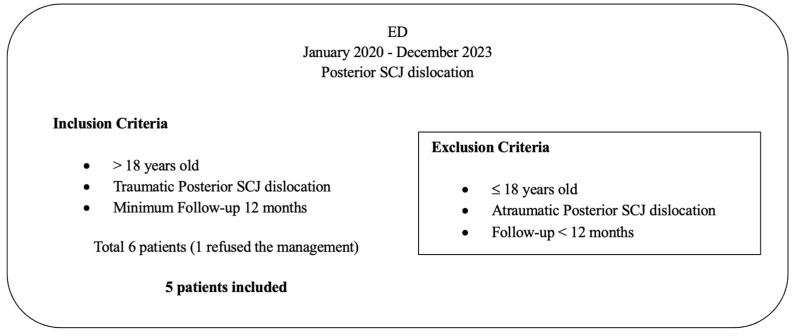
Inclusion and Exclusion Criteria.

All patients who were older than eighteen years old and presented with a traumatic posterior SCJ dislocation were included in the study, with no other exclusion criteria except for the follow-up. Indeed, patients who did not complete a minimum follow-up of 12 months and who had atraumatic posterior SCJ dislocation were excluded. This last group was excluded since the atraumatic dislocations follow other management pathways, and, as reported in the literature, they tend to occur in patients with different baseline characteristics, as in patients with trapezius palsy or generalized hyperlaxity [1]. Inclusion and exclusion criteria are summarized in Scheme 1.

Six patients who met the inclusion criteria were detected (five males and one female), but one of them was excluded because the patient refused the proposed treatment. Consequently, five patients, all males, were included in the study. The mean age was 34.4 years old (range 28–43 years old). All patients underwent a complete physical examination of the other near joints; three patients had not sustained any other injuries, two patients reported other fractures (one of them an ipsilateral distal fibular fracture, and the other a contralateral distal radial fracture, respectively). Three of them had a fall from a height, one patient reported a motor-bike accident and one had a trauma during sports activity, specifically during a rugby match. All of them reported indirect trauma, with a blow on the posterolateral side of the shoulder without loss of consciousness.

**Figure 1:**
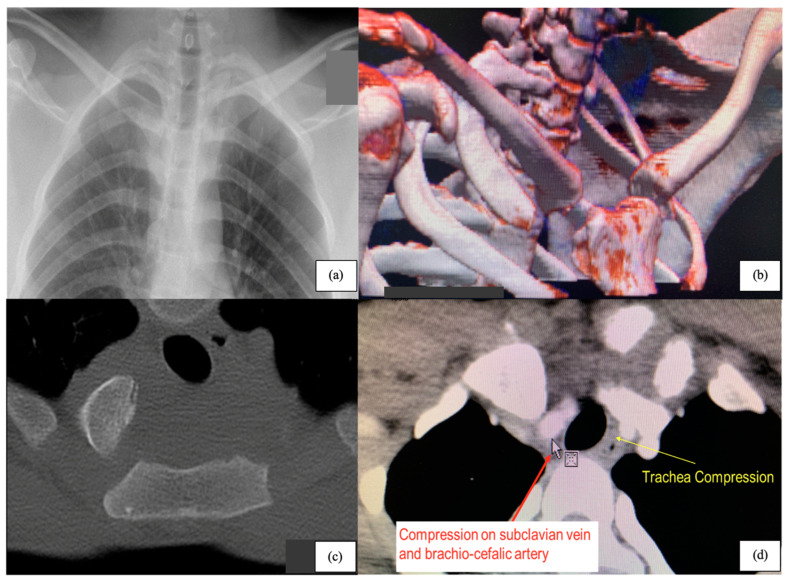
Preoperative evaluation of posterior SCJ dislocation: (**a**) chest X-ray; (**b**) 3D reconstruction; (**c**) axial CT scan bone view; (**d**) axial CT scan.

They were all subjected to X-ray examinations when they arrived at the ED. Moreover, a CT scan was requested for all cases to have a definitive diagnosis and for the potential involvement of thoracic essential organs. However, no systemic symptoms, such as cough, hoarseness, shortness of breath or venous congestion were present. At the CT scan, complete posterior dislocation of the SCJ was always confirmed (Figure 1). In addition, fractures and infractions of the ribs, lung parenchymal contusions and apical subpleural hematomas were detected.

A closed reduction maneuver was performed acutely in all cases in the ED under sedation and local anesthesia with the presence of the anesthesiologist and the vascular surgeon. After the trial maneuver, a CT scan was performed to assess the reduction. The reduction was fruitless in two cases, while in three patients it led to a reduction of the dislocation.

### 2.1. Conservative Treatment

For the three successful cases, once a CT scan verified the reduction, the patients were discharged home, and four outpatient visits were scheduled at 1–3–6–12 months after the trauma. At the follow-ups, X-rays were usually performed along with clinical examination, except for the third month where a CT scan was used to assess the maintenance of the reduction. A CT scan was also employed in cases of inconclusive X-rays. No recurrences were recorded in imaging, and, at the last follow-up, all patients had successfully recovered complete range of motion of the shoulder (in abduction/adduction, rotation and flexion/extension) and muscle strength, were pain-free while moving their arms and were back to their daily lives.

### 2.2. Surgical Treatment with a Figure-of-Eight Semitendinosus Tendon Autograft Reinforced with High-Strength Suture Tape

Regarding the two closely irreducible dislocations, the patients were admitted to the hospital for open surgical reduction and SCJ reconstruction. After the exclusion of SARS-CoV-2 infection through a nasopharyngeal swab in accordance with the COVID-19 protocol rules [20,21], the patients underwent surgery. The subsequent described procedure was the same for both patients.

A second closed reduction was attempted in the operating room, in the hopes anesthesia would simplify the procedure. The anesthesiologist was present throughout the procedure, and the thoracic surgeon and vascular surgeon were on standby in the operating room. Once the anesthesia was performed, we attempted the maneuvers described by Rockwood and Buckerfield maneuvers, without obtaining a successful reduction [1,14,22]. We tried, without success, to clamp the proximal end of the clavicle with bone forceps and pull and rotate it upwards. Consequently, an open reduction was needed. Given the detected instability, SCJ reconstruction with an autologous tendon graft in a figure-of-eight was selected. The medical team was made up of orthopedic surgeons, a thoracic surgeon and an anesthesiologist.

**Figure 2:**
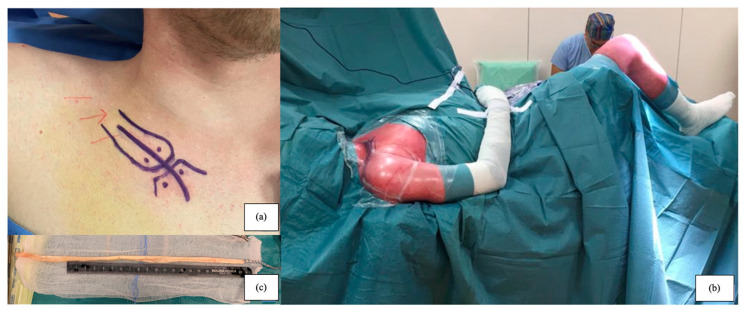
Surgical setting: (**a**) anatomical landmarks for identification of SCJ and drill hole planning; (**b**) double sterile field for SCJ access and tendon harvest; (**c**) tubularized autologous semitendinosus tendon graft.

The first surgical step was the harvest of the tendon graft. The semitendinosus tendon was preferred. The patients were positioned supine with a tourniquet to the thigh, and 2 g of cefazoline were administered preoperatively (Figure 2). For both surgeries, after setting up the sterile field, the tendon was harvested, tubularized with a non-absorbable suture on a workstation and then stored in a compound made of 500 mL of saline solution 0.9% + 1 g of vancomycin, similar to the hamstring harvest for anterior cruciate ligament reconstruction. The first surgical step ended with tourniquet removal, adequate hemostasis, surgical site washing, drain-positioning, suturing and dressing.

**Figure 3:**
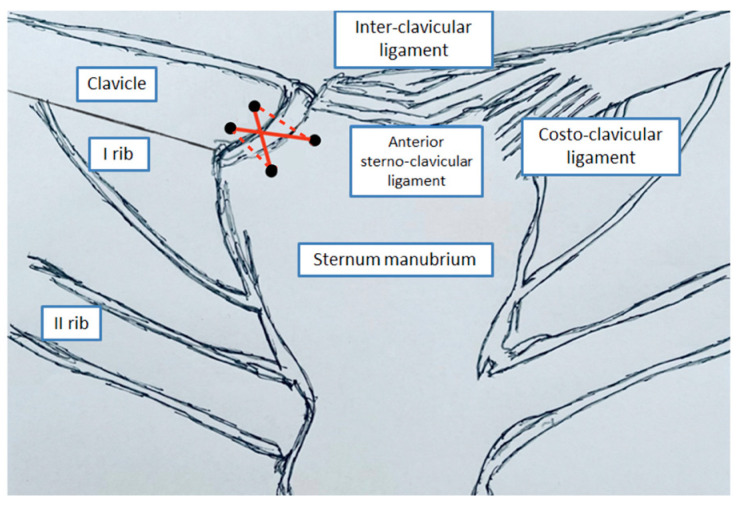
Preoperative planning.

In the second surgical stage, open reduction of the SCJ and subsequent stabilization with the autologous semitendinosus tendon graft in a figure-of-eight reinforced with high-strength suture tape was accomplished according to the preoperative planning (Figure 3).

**Figure 4:**
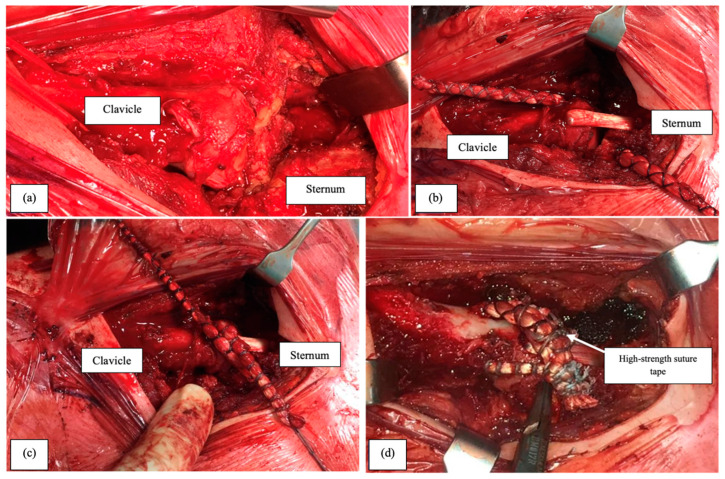
Intraoperative details of reconstruction with autologous semitendinosus tendon graft augmented with high-strength suture tape: (**a**) clavicle and sternum exposure; (**b**) graft passage in the drilled holes in the clavicle and sternum; (**c**) graft realization in the figure-of-eight; (**d**) figure-of-eight repair augmented with high-strength suture tape.

With the patient still supine in the sterile field, the SCJ was exposed with a straight incision extending from the center of the medial clavicle to the mid-superior aspect of the sternal manubrium (Figure 2a). The posterior SCJ dislocation was found. After releasing the medial part of the clavicle and the corresponding articular facet of the sternum, four bone tunnels of 5.0 mm in diameter were drilled, two in the clavicle and two in the sternum in parallel, adequately protecting the noble structures underlying these bones. After the reduction of the clavicle with blunt forceps, the joint was stabilized, with the semitendinosus tendon graft being passed through the holes in a figure-of-eight configuration and then reinforced with high-strength suture tape (Figure 4).

The post-operative protocol includes a neutral arm sling for the first three weeks after surgery, with active and passive movements forbidden. Elbow, wrist and hand mobilization were allowed and suggested from day one after surgery. The stitches were removed fifteen days after surgery. At three weeks, the patient started the rehabilitation of the shoulder with passive pendular movements first, and then with active motion in the fourth/fifth weeks. A return to sport was allowed six months after surgery. Both patients were followed up for one year postoperatively, specifically at 1–3–6–12 months after surgery. As for the conservative group, X-rays, along with clinical examination were usually performed at the follow-ups, except for the third month where a CT scan was used to assess the maintenance of the reduction. A CT scan was also employed in cases of inconclusive X-rays.

## 3. Results

All five patients, both the conservatively treated and the surgically managed, had returned to their daily lives by 12 months of follow-up.

For those conservatively managed no recurrences were recorded on imaging, and all the patients had successfully recovered their range of motion and muscle strength and were pain-free while moving their arms.

**Figure 5:**
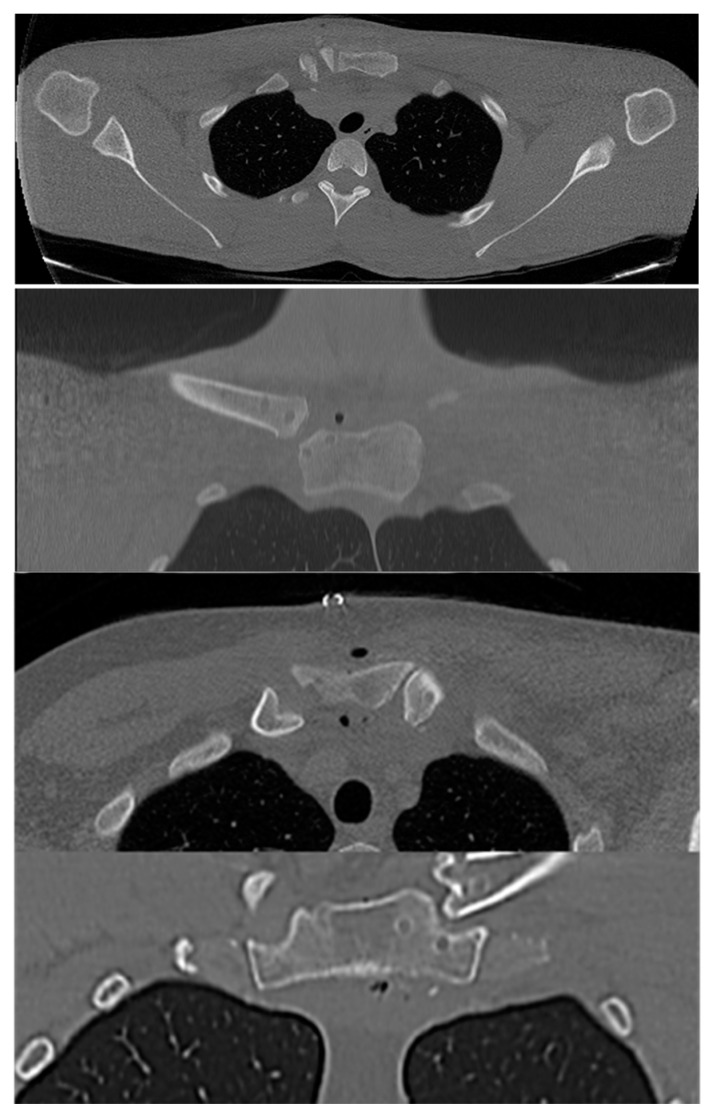
CT scan at 3 months post operation: reduction confirmed on axial and coronal scan on both patients.

**Figure 6:**
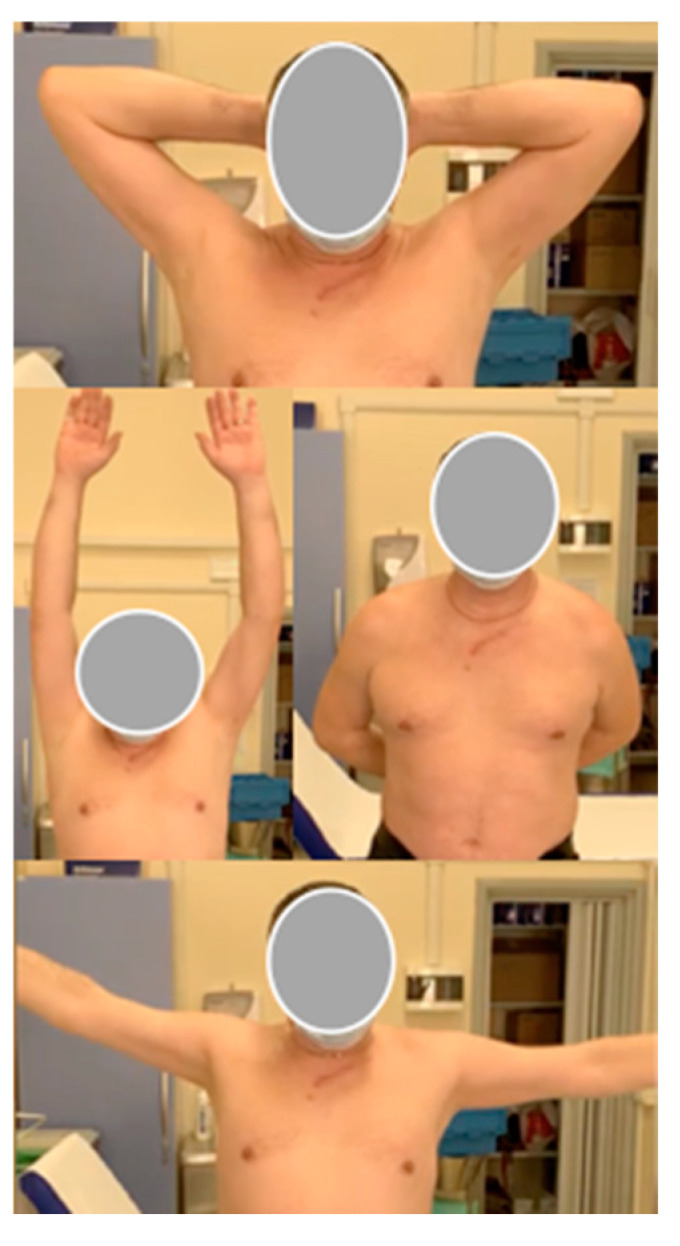
Clinical evaluation, 1 year follow-up, ROM restored and wound healed.

For those surgically treated, at three months the CT scan showed a complete reduction of the SCJ, both in the axial and the coronal views (Figure 5). At the end of the follow-up, ROM was restored, with an optimal strength recovery and no neurological deficits; in both cases, the healing was successful; no recurrences were recorded, and the patients returned to their daily lives without pain while moving their shoulder girdles (Figure 6).

A comprehensive comparison with the most recent literature will be provided in Section 4.

## 4. Discussion

This paper describes five cases of posterior traumatic SCJ dislocation handled according to the most recent recommendations, implementing the literature in this rare but potentially life-threatening pathology [1,2,3]. The diagnostic algorithm with the initial management and the surgical strategy adopted for the failed closed reduction cases align with the literature since SCJ reconstruction with tendon autograft and allograft is increasingly promoted due to biomechanical studies [1,2,3,19]. Indeed, reconstruction without fusion allows restoration of the ROM without the increased risk of developing degenerative changes or osteoarthritis of the distal joints that would otherwise need to compensate.

However, SCJ dislocation remains a rare pathology and the scientific literature about this topic is still poor, with the introduction of recommendations and systematic review only occurring in recent years [1,2,3].

The demographic characteristics, as well as the mechanism of trauma reported, are in line with the literature [1,2,3]. Kendal et al., in their systematic review, reported a mean age of 25.3 years old, with most patients being males [2]. Trauma is the most common cause of posterior SCJ dislocation, with high-energy processes in contact sports such as rugby and motor-vehicle collisions being the most common culprits [5,6,12].

They result either from indirect trauma when a posterolateral compressive force is applied to the shoulder and transmitted to the SCJ as a vector force or as direct anteroposterior trauma to the SCJ [1,2,3]. Atraumatic SCJ dislocations exist, but they tend to occur in patients with generalized hyperlaxity or trapezius palsy [1,2].

Due to the anatomy of the SCJ, which has a robust posterior capsular ligament and the costoclavicular ligament, anterior dislocation occurs more frequently, with posterior SCJ dislocations comprising 5% to 27% of all SCJ dislocations [15,22].

From a clinical point of view, posterior SCJ dislocation is often characterized by neck and shoulder pain increased by ipsilateral shoulder movement [1,3,9]. A deformity of the SCJ can be present even if swelling of the joint gives the illusion of an anterior dislocation in the case of a posterior dislocation [1,14]. As reported by Ingoe et al. it is advisable to examine the nearby joints, such as the acromioclavicular joint (ACJ), in order to detect other injuries which could lead to a floating clavicle [3,23,24]. Posterior SCJ dislocation should be suspected and identified since it can lead to life-threatening complications due to the presence of noble structures near the joint: disorders of the neurological structures (brachial plexus, phrenic and vagus nerves) and/or vascular ones (compression or laceration of the subclavians, internal jugulars, internal thoracic or brachiocephalic vessels), pneumothorax, myocardial conduction abnormalities, esophageal or tracheal rupture, mediastinal compression and thoracic outlet syndrome could occur [2]. In case of complications, wider symptomatology, such as the presence of cyanosis, choking, cervical bruit, tracheal hematoma, stridor, dyspnea and respiratory distress, dysphagia, hoarseness, odynophagia, compromised circulation to the arm, diaphragmatic paralysis and even death could occur [7,8,9,10]. Overlapping adjacent structures make assessing and interpreting SCJ dislocation on routine chest radiographs difficult [1,2,3]. SCJ dislocation may not be so immediate to diagnose, even with dedicated radiographic views, such as the serendipity view [2,7,14]. Moreover, the literature agrees about distinguishing between SCJ dislocation and physeal fractures, since the medial clavicular physis is the last to close around 23–25 years of age [9,12,15]. Consequently, a computed tomography (CT) scan is the imaging modality of choice for definitive diagnosis and a three-dimensional understanding of the SCJ [1,2,3,9]. When vascular injuries are suspected, intravenous contrast can be administered [1,2,3]. In the cases described, a CT scan was performed pre- and post- operatively and post-reduction maneuvers to assess the maintenance of the reduction. MRI can be helpful in physeal injuries and in case of chronic instability to assess the ligament status [1,3,9]. In MRI the articular surfaces and the intra-articular disc are better visualized in coronal sequences, whereas axial sequences depict anterior and posterior capsules and ligaments [9]. The sagittal sequences are useful in assessing the costoclavicular ligament [9].

Recommendations from the literature agree that posterior dislocation needs to be reduced when diagnosed [1,2,3,22].

A closed reduction should be attempted in the first 48 h, with a success rate reported in adults by Ingoe et al. between 38% and 50% [3,24]. If this maneuver is effective, a follow-up CT scan can be indicated to detect any recurrent dislocations [1,2,3]. On the other hand, both the failure of closed reduction maneuvers and the frequent presentation over 48 h, due to the aforementioned difficulties in diagnosis, require an open reduction [1,2,3,22]. Indeed, a closed reduction after 48 h has a high probability of being unsuccessful [2,18]. The five cases described in this study underwent this management algorithm, with a first closed reduction acutely attempted.

Moreover, the literature suggests that when closed reduction is successful, the SCJ is usually stable without additional intervention, while in case of failure and open reduction, the SCJ needs to be reconstructed [1,2,3]. However, a universally accepted stabilization method for posterior SCJ dislocation does not exist, and various techniques have been described depending on several features, such as biomechanics, the patient’s future expectation goals, surgical expertise and degree of instability [1,2,3].

### 4.1. Anatomy and Biomechanics of the SCJ

The SCJ is the only true connection between the upper limb and the axial skeleton, representing one of the most stable joints in the human body [1,2,3,9]. It is a diarthrodial joint with a saddle-like shape where only the lower 2/3 of the medial clavicle is wrapped by articular cartilage [1,2,25]. Due to the limited osseous congruence, SCJ stability lies on strong ligament structures and a fibrocartilaginous intra-articular disk, which has mobility along the anteroposterior and vertical axes [9,25]. The ligaments of the SCJ are represented by the anterior and the posterior sternoclavicular capsular ligament, the costoclavicular ligament and the interclavicular ligament [1,2,9,13,25]. The thicker posterior capsular ligament was considered in a biomechanical study by Spencer et al. to be the most crucial structure to avoid anterior and posterior translation, with the anterior capsular ligament impeding only anterior translation [1,9,25]. This is the main reason why most dislocations occur anteriorly since posterior SCJ dislocations require higher forces [1,2,25].

However, the costoclavicular ligament has recently received more attention [1,9,25]. It is a stout, short, flattened, inverted cone with two laminae on the anterior and posterior aspects of the clavicle [9,25]. Raising and lowering movements occur between the articular disk and the clavicle, whereas protraction and retraction occur between the articular disc and the sternum [1,2,3,9].

SCJ injury impacts the scapulothoracic and glenohumeral rhythm [1,2,3,9]. Most of the motion of the SCJ occurs in the anteroposterior direction with protraction and retraction of approximately 35° in either direction [1,2,3,26]. The SCJ can elevate up to 35°, and other essential movements are anterior and posterior rotation concerning the lateral axis of the clavicle [1,2,3,26].

For these reasons, the reconstruction procedure of the SCJ is essential to restoring the ROM of the shoulder girdle [1,2,3]. Recent literature suggests avoiding open reduction and internal fixation with plates and screws since this could limit glenohumeral motion and usually necessitate implant removal to restore function and avoid chronic degenerative changes [1,2,3].

Apart from the anatomy of the SCJ, it is important to mention the “safe zone” avascular plane behind the joint and anterior to the muscle belly, especially when reconstruction techniques requiring drilling holes are performed, since protective retractors could be placed in this location [1,2,3]. Finally, as mentioned above, since the medial clavicle is the last ossification center to fuse between the ages of 22 and 25, proximal clavicle physeal fracture-separation in young patients could mimic a sternoclavicular dislocation in up to 50% of cases [1,2,3,10,12]. As a result, these two entities should be distinguished [1,2,3].

### 4.2. Open Reduction and SCJ Stabilization and Reconstruction Options

Open reduction is needed when closed reduction fails and in cases of recurrent dislocation or chronic instability, and the literature advocates stabilization with SCJ reconstruction [1,2,3,19]. Indeed, as reported by Ingoe et al. direct repair of the ligaments is usually not achievable and reconstruction with sutures or tendons is advisable [3].

SCJ dislocation can be further classified as acute or chronic [1,2,14,15,16]. Kendal et al. reported in their systematic review that most are chronic dislocations resulting from missed injuries, delayed clinical presentation or recurrent dislocations following a closed acute reduction maneuver [1,2,14,15,16]. Moreover, Glass et al. stated that the effectiveness of the surgery is not adversely affected by failure of conservative treatment [27].

The presence in the operating theater of a vascular or cardiothoracic surgeon and an anesthesiologist is usually endorsed since teamwork and a shared approach leads to safer surgery [1,2,3]. The two reported cases were managed according to the abovementioned suggestions, and the equipped specialized surgical team was present in the operating room.

A universally accepted stabilization method does not exist, and the literature reports many options [1,2,3,22,28]. However, recently, recommendations have been developed [1,2,3]. Still, results are hard to compare because studies are often characterized by small numbers of patients and different treatment options (such as internal fixation with plates and screws, cannulated screws, trans-osseous suture, reconstruction with hamstring tendons or LARS, and tension bands) and by a short follow-up. In addition, few of them differentiate acute from chronic SCJ dislocation; therefore, advantages and disadvantages for each technique are reported [1,2,3,28].

The literature agrees that open reduction and internal fixation with screws and plates should be avoided unless in the case of comminuted fracture-dislocations [1,2,3]. This is because implants usually need to be removed since they lead to an arthrodesis, thus limiting motion on the ipsilateral shoulder girdle with the possibility of degenerative changes [1,2,3]. Moreover, usually, a bi-cortical fixation is necessary, with the risk of screw thread protrusion posterior to the clavicle, where the vessels are located [3].

Pins or Kirschner wires should be avoided due to their possible migration, which can damage noble structures [29]. In particular, a risk of death of up to 40% has been estimated when using Kirschner wires due to potential vascular complications [9,19]. The most striking case in this regard was reported by Ballas et al. concerning a posterior SCJ dislocation treated with three wires: the first was removed after a short time since it was protruding below the skin; the second protruded vertically in front of the sternum after two years, giving symptoms similar to myocardial infarction; the last one migrated into the pelvic cavity giving abdominal symptoms, and multiple surgical interventions were necessary to extract it [29]. In the same paper, the authors also carried out a literature review, reporting 88 cases of wire migration, of which 18 followed sternoclavicular joint dislocation [29].

Another surgical solution is medial clavicular resection with soft tissue reconstruction [1,2]. This is usually reserved for cases with degenerative changes, medial clavicle fractures or irreducible locked dislocations [1,2].

Thus, the literature conveys that SCJ reconstruction should be adopted with biological (such as allograft or autograft) or synthetic grafts [1,2,3,28]. Indeed, both Provencher et al. [1] and Kendal et al. [2] suggested the use of a figure-of-eight tendon autograft or allograft. This configuration has gained popularity after the biomechanical study by Spencer and Kuhn which evaluated three different reconstruction techniques with cadaveric models [19]. They assessed (1) the intramedullary ligament reconstruction, (2) the subclavius tendon reconstruction and (3) the use of a figure-of-eight of a semitendinosus graft through drill holes in the clavicle and the sternum’s manubrium [19]. They demonstrated that the figure-of-eight graft’s stiffness and load to failure were greater than the other two techniques, especially in the posterior direction [19].

However, this procedure is not without complications and tips are given by several authors [1,2,3,19]. The drill holes in the bones should be spaced 1.0 to 1.5 cm apart to allow for adequate bone ridge and 1.0 to 2.0 cm away from the end of the clavicle to avoid stress fracture [1,2,19]. The graft type usually guides the size of the holes [1].

Several grafts are used, such as the palmaris, the semitendinosus and the gracilis, with overall good outcomes and a low revision rate [1,2,3].

The authors of this paper, in agreement with the literature, chose the figure-of-eight configurations for one crucial reason: despite the SCJ having a limited range of motion, this joint is fundamental for the physiology of the shoulder girdle and the resulting stabilization with ORIF would fix and limit its ROM. Subsequently, stress concentration may occur during functional exercises, and the surgical fusion of the joint could induce the patient to compensate for any movement by using the downstream joints [1,2,3,9]. Indeed, the paper’s authors believe that SCJ ROM should be re-established in order to avoid chronic degenerative changes to it or to the downstream joints.

In this regard, Tytherleigh-Strong et al. evaluated a series of 19 patients with acute traumatic posterior SCJ dislocation treated with hamstring tendon autograft reconstruction technique within 14 days from the injury [30]. After a minimum follow-up of three years, they reported a high survivorship grade (96%), good clinical outcomes and a high rate of return to sports, since 86% of patients who practiced sports were able to return to their pre-injury level [30]. In addition, Provencher et al. analyzed patients operated with a figure-of-eight technique, reporting a revision rate of 8.3% for recurrent or persistent instability [1].

Using autologous tendons increases the risk of morbidity at the graft-harvest site as well as infection associated with the use of the graft tissue, which, however, are rarely reported [1,2,3]; conversely, it removes the intolerance to osteosynthesis devices and graft rejection [1,2,3]. The authors did not report complications, neither at the graft site nor at the SCJ surgical site.

Artificial ligaments are another similar surgical way to stabilize the SCJ in young and active patients [28]. The employment of the LARS (Ligament Augmentation and Reconstruction System) technique has been demonstrated to be a feasible option, able to address both the costoclavicular and capsular ligaments [28]. Quayle et al. reported 5 cases of SCJ dislocations treated with LARS and interference screws, with promising short- and mid-term outcomes with lowering of pain and improvement of joint function [28]. However, in all these reconstruction procedures, fusion of the joint is avoided, and SCJ motion is restored [1,2,3].

Lastly, even if repair is usually not achievable [3], Kendal et al. proposed in acute cases the use of synthetic material such as sutures passed through uni-cortical holes to repair and augment the SC ligament, avoiding damage to the mediastinal structures and preventing iatrogenic physeal injuries in children [2].

In the cases presented in this work, a high-strength suture tape was used as an internal brace as described by Ingoe et al. [3].

This study, with its strengths and limitations, is significant in that it aligns with the current literature. It does so by following the recommendations and adopting the most suggested surgical technique.

However, it includes few patients and a one-year follow-up.

Moreover, since the number of participants is limited, a descriptive analysis of both the surgical technique and the results with a comparison with the current literature was mainly performed. A statistical analysis was not accomplished.

As mentioned, this is a common limitation in the literature since this pathology is rare, and it is difficult for a single center to have a huge cohort of patients.

Consequently, for future research, higher-quality studies, especially multicenter studies with control groups or RCTs, will be necessary to determine the best surgical technique.

## 5. Conclusions

Traumatic posterior SCJ dislocation can lead to life-threatening consequences due to its proximity to vital structures. After diagnosis, a closed reduction in the first 48 h is suggested, and, in case of failure, open reduction and SCJ reconstruction are indicated. No universal guidelines for the stabilization method exist, but the recent literature suggests a figure-of-eight reconstruction with a tendon allograft or autograft. A series of posterior traumatic SCJ dislocations is reported, managed according to the recent recommendations either conservatively or surgically with a figure-of-eight semitendinosus tendon autograft augmented with high-strength suture tape. Given the importance of SCJ motion for the shoulder girdle, the authors of this paper adopted this technique, and they reported, at one year of follow-up, a complete shoulder ROM with full strength throughout.

## Data Availability

The original data presented in this study are available under request to the corresponding author.

## Abbreviations

SCJ	sternoclavicular joint
ROM	range of motion
g	gram
ED	Emergency department
CT	computed tomography
MRI	magnetic resonance imaging
LARS	ligament augmentation and reconstruction system
ORIF	open reduction and internal fixation
RCTs	randomized controlled trials
mm	millimeters
ml	milliliters
ACJ	acromioclavicular joint
